# Risk factors for avian influenza virus in backyard poultry flocks and environments in Zhejiang Province, China: a cross-sectional study

**DOI:** 10.1186/s40249-018-0445-0

**Published:** 2018-06-19

**Authors:** Xiao-Xiao Wang, Wei Cheng, Zhao Yu, She-Lan Liu, Hai-Yan Mao, En-Fu Chen

**Affiliations:** Zhejiang Provincial Centre for Disease Control and Prevention, 3399 Binsheng Road, Binjiang District, Hangzhou, Zhejiang 310051 People’s Republic of China

**Keywords:** Risk factor, Avian influenza virus, Backyard poultry, Environmental contamination

## Abstract

**Background:**

Human infection of avian influenza virus (AIV) remains a great concern. Although live poultry markets are believed to be associated with human infections, ever more infections have been reported in rural areas with backyard poultry, especially in the fifth epidemic of H7N9. However, limited information is available on backyard poultry infection and surrounding environmental contamination.

**Methods:**

Two surveillance systems and a field survey were used to collect data and samples in Zhejiang Province. In total, 4538 samples were collected by surveillance systems and 3171 from the field survey between May 2015 and May 2017, while 352 backyard poultry owners were interviewed in May 2017 by questionnaire to investigate factors influencing the prevalence of avian influenza A virus and other AIV subtypes. RT-PCR was used to test the nucleic acids of viruses. ArcGIS 10.1 software was used to generate maps. Univariate and logistic regression analyses were conducted to identify risk factors for AIV infection.

**Results:**

Of the 428 poultry premises observed by the surveillance system, 53 (12.38%) were positive for influenza A virus. Of the 352 samples from poultry premises observed by field survey, 13 (3.39%) were positive for influenza A virus. The prevalence of AIV was unevenly distributed and the dominant subtype differed among cities. Eastern (Shaoxing and Ningbo) and southern (Wenzhou) cities exhibited a higher prevalence of AIV (16.33, 8.94, and 7.30% respectively). Contamination of AIV subtypes was most severe in January, especially in 2016 (23.26%, 70/301). The positive rate of subtype H5/H7/H9 was 2.53% (115/4538). Subtype H5 was the least prevalent, while subtypes H7 and H9 had similar positivity rates (1.50 and 1.32% respectively). Poultry flocks and environmental samples had a similar prevalence of AIV (4.46% vs 5.06%). The type of live birds was a risk factor and the sanitary condition of the setting was a protective factor against influenza A contamination.

**Conclusions:**

AIV subtypes were prevalent in backyard poultry flocks and surrounding environments in Zhejiang Province. The types of live birds and sanitary conditions of the environment were associated with influenza A contamination. These findings shine a light on the characteristics of contamination of AIV subtypes and emphasize the importance of reducing AIV circulation in backyard poultry settings.

**Electronic supplementary material:**

The online version of this article (10.1186/s40249-018-0445-0) contains supplementary material, which is available to authorized users.

## Multilingual abstracts

Please see Additional file 1 for translations of the abstract into the six official working languages of the United Nations.

## Background

Avian influenza virus (AIV), which has given rise to multiple genotypes, poses an unprecedented public health threat and has brought about huge economic losses to society. The H5N1 subtype of AIV, which was first detected in Guangdong Province, China in 1996, has resulted in 859 confirmed human infections in more than 60 countries as of June 15, 2017 and continues to cause poultry outbreaks from time to time [1, 2]. The H7N9 subtype of AIV, which was first reported in Shanghai, China in 2013 [3], has resulted in more than 1500 cases of human infection in 26 provinces in China. In November 2013, the first human infection of a novel avian-origin reassortant of H10N8 virus was reported in Jiangxi Province, China [4]. Since first isolated from wild birds and poultry in Italy, Australia, Sweden, Canada, USA, China, Korea, and Japan in 1965 [5, 6], the spread of the H10N8 virus from poultry to humans has become a public health concern worldwide. Besides, multiple subtypes (H7N7 [7], H5N6 [8], H6N1 [9], H9N2 [10], H10N7 [11], etc.) have also resulted in repeated human infections in Asia, Europe, Africa, and North America since 1956.

It has been demonstrated that contamination of poultry-related environments, including live poultry markets (LPMs) and backyard poultry flocks, is a major risk factor for human AIV [12, 13]. Previous studies have reported differences in the poultry exposure to AIV between urban and rural areas. In urban areas, LPMs are the primary sources of human AIV infection, while backyard poultry flocks are the major causes of exposure in rural environments [14]. As an effective measure to prevent AIV exposure, LPMs have been closed in many provinces of China. Consequently, human cases of H7N9 infection have gradually spread from urban to semi-urban and rural areas [15]. Zhejiang Province of China, which reportedly has the greatest number of human cases of H7N9 infection, also experienced a significant increase in the number of rural infections from 2013 to 2017 [16]. Given the poor knowledge, attitudes, and practices of backyard poultry holders [17, 18], the prevention and control of human AIV infections from backyard poultry flocks has created a great challenge to the governments of rural areas.

Surveillance systems provide evidence-based data to understand the temporal and spatial distributions of viruses in poultry and environments, so that targeted measures can be implemented to prevent viral transmission to humans [19, 20]. Based on these premises, Zhejiang Province began environmental surveillance of AIV in non-commercial backyard poultry flocks in 2011 [21, 22]. Given the increase in human H7N9 infections in rural areas, we implemented a surveillance system to monitor the prevalence of AIV in samples collected from backyard poultry flocks and surrounding environments from May 2015 to May 2017 [23]. Further, in order to elucidate the risk factors for contamination of backyard poultry flocks and surrounding environments, a structured questionnaire was designed to collect information from backyard poultry breeders. This study presents the results of the surveillance system and field survey to provide clues to improve surveillance and proposes a strategy for disease prevention and control.

## Methods

### Surveillance system and sample collection

**Fig. 1:**
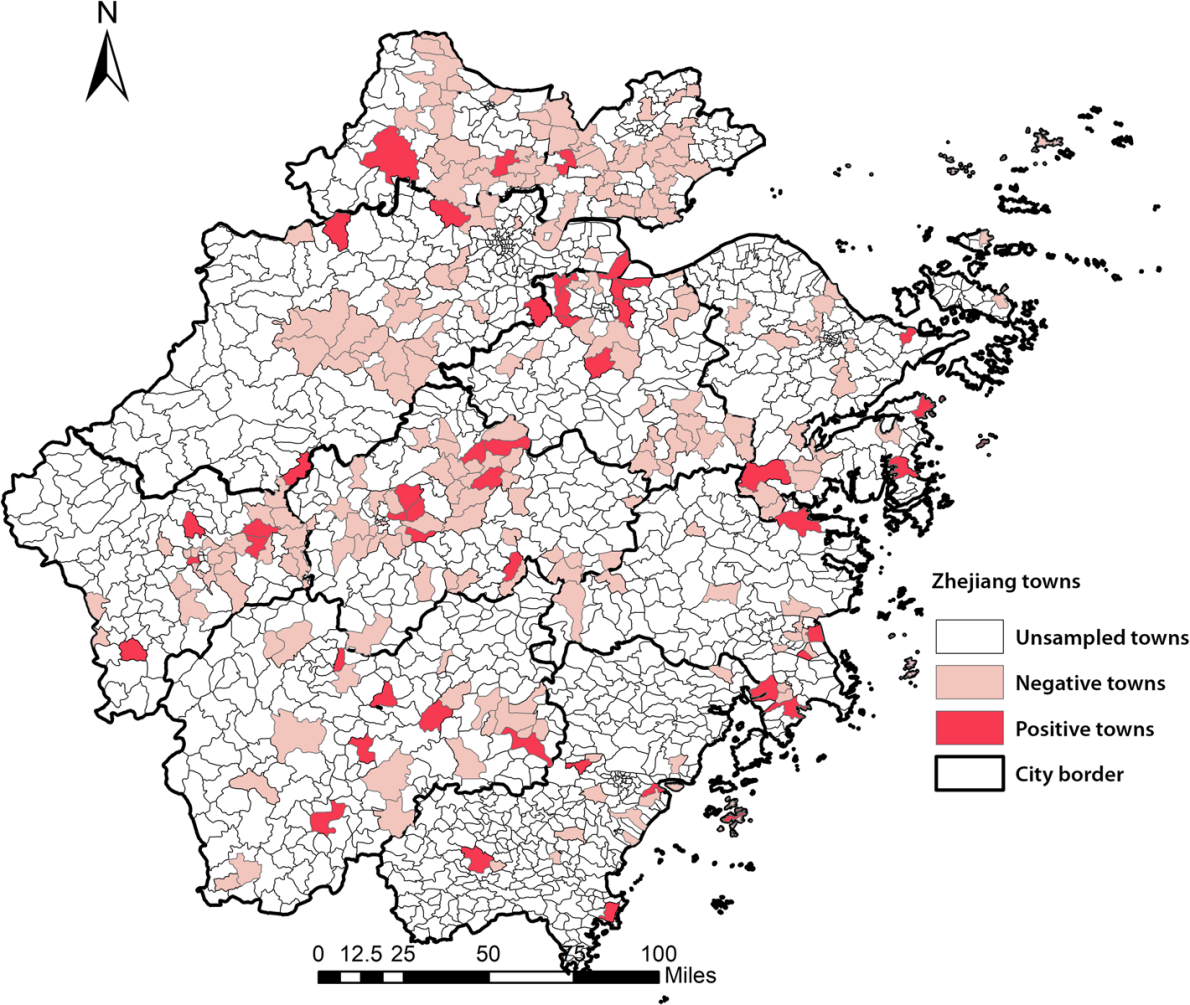
Distribution of avian influenza virus -positive towns and -negative towns in Zhejiang Province

Two different surveillance systems were implemented in Zhejiang Province. The surveillance design and sample sites during epidemic periods (October–March) are described in our previous study [23]. A total of 30 samples were collected each month from each of 11 cities (Hangzhou, Ningbo, Wenzhou, Jiaxing, Huzhou, Shaoxing, Quzhou, Jinhua, Taizhou, Lishui and Zhoushan) in Zhejiang Province. Between April and September, 15 samples were obtained once a month from each city. Sampling sites included LPMs, backyard poultry flocks, poultry rearing farms, slaughtering and processing facilities, and wild bird habitats. In this study, we analyzed the specimens collected from backyard poultry flocks and surrounding environments from 11 cities in Zhejiang Province between May 2015 and May 2017. The positive rate was calculated at the city level (Additional file 2: Table S1) and at the town level (Fig. 1). Further, to present more detailed information of AIV spatial distribution, data analysis was also calculated at the premises level.

Briefly, of 1523 towns in Zhejiang, 255 (16.74%) were sampled in our study between May 2015 and May 2017. A total of 4538 samples were collected from 428 backyard poultry premises. Specifically, 2778 samples were from poultry flocks (including 2734 feces, 14 anal swabs, 16 throat swabs, and 14 feather swabs) and 1760 from environments (including 814 poultry cage swabs, 648 drinking water samples, 122 crib swabs, 98 sewage samples, 50 environmental surface swabs, and 28 chopping board swabs).

### Field survey

In addition to the surveillance systems, a cross-sectional survey was conducted among backyard poultry breeders in May 2017 to investigate the quantity of flocks, the sources of poultry (LPMs, illegal itinerant vendors, as a present from friends or relatives, hatching), flock health history, method of disposal of sick and dead birds, flock vaccination history, cleaning and disinfection practices of the premises, sanitary conditions of the premises, type of nesting area (enclosed or open), and mode of raising birds (free range or captivity). Backyard poultry breeders were selected based on the county level to achieve a balanced spatial distribution throughout the province. Namely, four backyard poultry breeders from each county were invited to participate in the study. Premises without backyard poultry or poultry owners unwillingly to provide information were excluded. A total of 352 backyard poultry breeders were interviewed in person by trained epidemiologists in May 2017. At the same time, as a supplement to the surveillance system, a total of 3171 samples were collected from backyard poultry flocks and environments (9 or 10 from each backyard poultry premise).

### Sample transportation and laboratory testing

All specimens collected from the surveillance systems and field survey were stored at 4 °C and transported to the local Center for Disease Control and Prevention (CDC) within 48 h. RNA of avian influenza A virus was extracted from all specimens using the RNeasy Mini Kit (Qiagen, Hilden, Germany) and tested with the QuantiTect™ Probe RT-PCR Kit (Qiagen, Hilden, Germany), according to the manufacturer’s instructions using the following primers and probes: FluA-forward (5′-GAC CRA TCC TGT CAC CTC TGA C-3′), FluA-reverse (5′-GGG CAT TYT GGA CAA AKC GTC TAC G-3′), and FluA-probe (5′-TGC AGT CCT CGC TCA CTG GGC ACG-3′). Given that H5, H7, and H9 were the most prevalent subtypes affecting human beings, influenza A positive specimens were further subtyped for H5, H7, or H9. The primers, probes, and RT-PCR conditions for detection of subtypes H5, H7 and H9 are previously reported [24]. The results were validated by the Zhejiang CDC. However, viral isolation was not conducted in this study. Notably, specimens identified as subtype H5, H7, or H9 were categorized as “H5/H7/H9 positive.”

### Data analysis

A database of the questionnaire responses was constructed using EpiData software (version 3.0, EpiData Association, Denmark, http://www.epidata.dk/). All statistical analyses were performed using IBM SPSS Statistics for Windows (version 21.0, IBM Corp., Armonk, NY, USA). Maps of the sampling sites and prevalence of AIV in cities were generated with ArcGIS 10.1 software (ERSI Inc., Redlands, CA, USA http://www.esri.com/arcgis/about-arcgis). Categorical variables were analyzed using the chi-squared test, while the Fisher exact test was applied when appropriate. Logistic regression analysis was used to analyze multiple risk factors. The criterion for variables included for multiple risk analysis was a probability (*P*) value of < 0.1 and additional variables with biological plausibility. Stepwise variable selection was performed for logistic regression analysis. Variables with a *P*-value of < 0.05 were considered significant in all statistical analysis. Poultry quantity was redefined as a categorical variable when analyzing risk factors, while the subgroup was divided according to the mean (μ = 19).

## Results

### General information

Of the 428 poultry premises observed by the surveillance system, 53 (12.38%) were positive for avian influenza A. Of the 4538 samples, 213 (4.69%) were found to be positive for avian influenza A (Table 1). In addition, the positive rate of subtype H5/H7/H9 was 2.53% (115/4538). Subtype H5 was the least prevalent, while subtypes H7 and H9 had similar positivity rates (1.50 and 1.32%, respectively).

**Table 1:** Prevalence of avian influenza virus in different types of samples from surveillance system

Types	Sum	Prevalence (%)				
		A	H5	H7	H9	H5/H7/H9
Poultry samples	2778	124 (4.46)	6 (0.22)	21 (0.76)	36 (1.30)	57 (2.05)
Feces	2734	110 (4.02)	6 (0.22)	19 (0.69)	28 (1.03)	49 (1.79)
Anal swabs	14	2 (14.29)	0 (0)	0 (0)	0 (0)	0 (0)
Throat swabs	16	6 (37.5)	0 (0)	0 (0)	6 (37.5)	6 (37.5)
Feather swabs	14	6 (42.86)	0 (0)	2 (14.29)	2 (14.29)	2 (14.29)
Environment samples	1760	89 (5.06)	3 (0.17)	47 (2.67)	24 (1.36)	58 (3.30)
Cage swabs	814	43 (5.28)	3 (0.37)	18 (2.21)	13 (1.60)	23 (2.83)
Environmental surface swabs	50	9 (18.00)	0 (0)	5 (10.00)	4 (8.00)	9 (18.00)
Drinking water samples	648	14 (2.16)	0 (0)	7 (1.08)	5 (0.77)	7 (1.08)
Sewage samples	98	9 (9.18)	0 (0)	6 (6.12)	2 (2.04)	8 (8.16)
Crib swabs	122	0 (0)	0 (0)	0 (0)	0 (0)	0 (0)
Chopping board swabs	28	14 (50.00)	0 (0)	11(39.29)	0 (0)	11(39.29)
Total	4538	213 (4.69)	9 (0.20)	68 (1.50)	60 (1.32)	115 (2.53)
*χ*^2^	–	0.8474	–	26.7553	0.0379	6.7464
*P*-value	–	0.3573	1.000^a^	< 0.0001	0.8457	0.0094

^a^ Fisher exact test

Of the 352 poultry premises from the field survey, 13 (3.39%) were positive for avian influenza A and 3 (0.85%) were positive for subtype H9. Of the 3171 samples, 52 (1.64%) were positive for avian influenza A (Table 2) and 4 (0.13%) were positive for H9, while all samples were negative for subtypes H5 and H7. In general, the prevalence of all AIV subtypes was lower in the field survey than that from the surveillance systems.

**Table 2:** Prevalence of avian influenza virus in different types of samples from field survey

Types	Sum	Prevalence (%)				
		A	H5	H7	H9	H5/H7/H9
Poultry samples	16 10	21 (1.30)	0 (0)	0 (0)	0 (0)	0 (0)
Feces	16 10	21 (1.30)	0 (0)	0 (0)	0 (0)	0 (0)
Environment samples	1561	31 (1.99)	0 (0)	0 (0)	4 (0.26)	4 (0.26)
Cage swabs	585	7 (1.20)	0 (0)	0 (0)	3 (0.51)	3 (0.51)
Drinking water samples	519	9 (1.73)	0 (0)	0 (0)	0 (0)	0 (0)
Sewage samples	82	0 (0)	0 (0)	0 (0)	0 (0)	0 (0)
Crib swabs	294	13 (4.42)	0 (0)	0 (0)	0 (0)	0 (0)
Environmental surface swabs	81	2 (2.47)	0 (0)	0 (0)	1 (1.23)	1 (1.23)
Total	3171	52 (1.64)	0 (0)	0 (0)	4 (0.13)	4 (0.13)
*χ*^2^	–	2.2825	–	–	–	–
*P*-value	–	0.1308	–	–	–	–

### Spatial distribution of avian influenza a and subtypes in backyard poultry

**Fig. 2:**
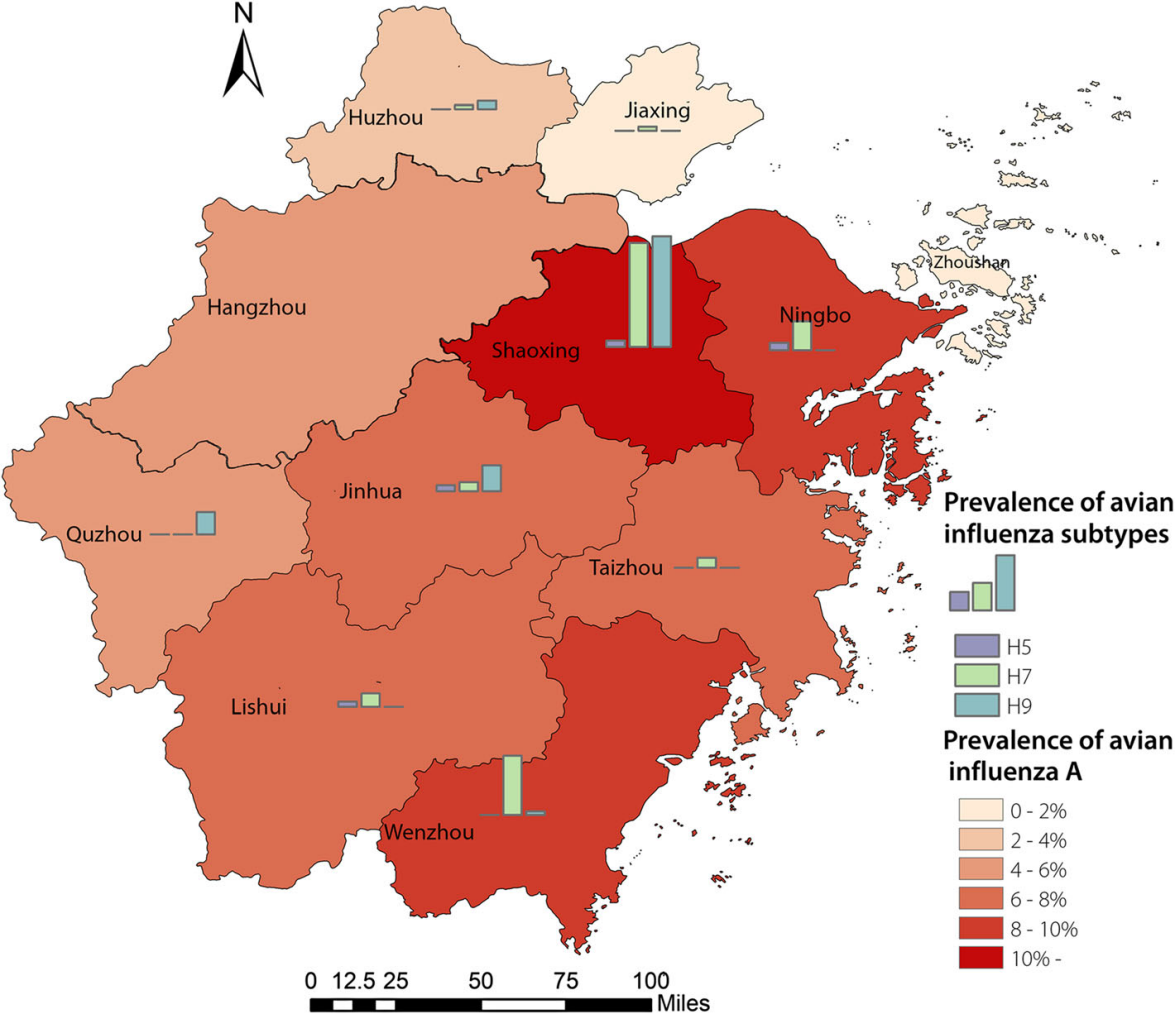
Prevalence of avian influenza subtypes in different cities from 2015 to 2017

As shown in Fig. 2 and Additional file 2: Table S1, the prevalence of avian influenza A was unevenly distributed in Zhejiang Province at the city level. Further, the dominant subtype differed among cities. The positive rate of avian influenza A was overwhelmingly greater in Shaoxing (16.33%, 65/398) than that in other cities, as was also the case for subtypes H7 (8.04%, 32/398) and H9 (8.54%, 34/398). However, in Zhoushan, all samples were negative for contamination. Ningbo city had a relatively higher proportion of samples that were positive for avian influenza A (8.94%, 16/179). Besides, in Jiaxing, Lishui, Taizhou, and Wenzhou, H7 was the dominant subtype, while in Quzhou and Jinhua, H9 was the dominant one. Figure 1 shows the prevalence of avian influenza A at the town level. Of the 255 towns, 46 (18.04%) were contaminated.

### Temporal distribution of avian influenza A and subtypes in backyard poultry flocks

**Fig. 3:**
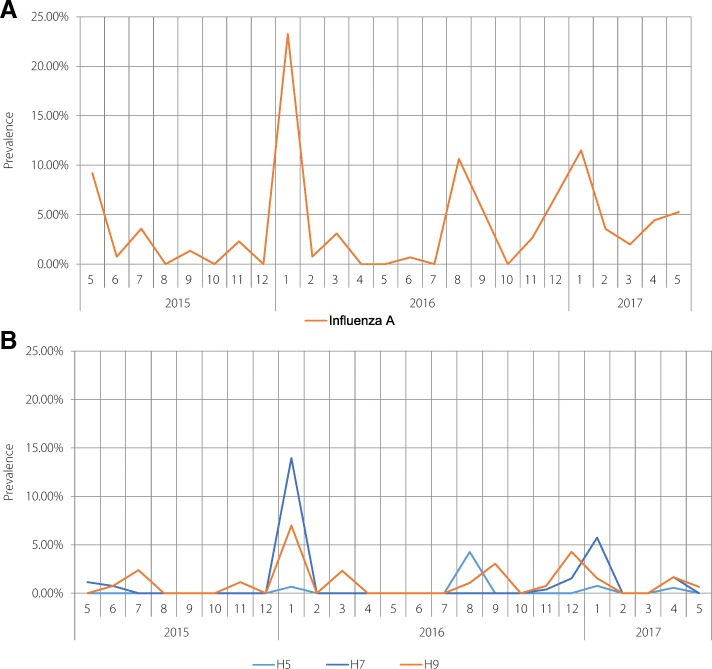
**a**. Prevalence of avian influenza A in backyard poultry and environments from 2015 to 2017. **b**. Prevalence of avian influenza subtypes (H5, H7, H9) in backyard poultry and environments from 2015 to 2017

The trends of the temporal distributions of avian influenza A and subtypes are shown in Fig. 3. The prevalence of avian influenza A peaked in January 2016 (23.26%, 70/301), with relatively high frequencies in August 2016 and January 2017 (10.64%, 10/94 and 11.49%, 30/261, respectively). The prevalence of subtype H7 peaked in January 2016. Positive samples for subtype H5 were only found in January and August 2016, and January and April 2017. The highest level of subtype H5 occurred in August 2016 (4.26%, 4/94). In addition, the temporal distribution of subtype H9 was similar to that of avian influenza A. The contamination of AIV subtypes was most severe in January, especially in 2016.

### Distribution of avian influenza A and subtypes in different sample types

As shown in Table 1, there was no significant difference in the prevalence of AIV subtypes between the poultry and environmental samples, with the exception that subtype H7 was more prevalent in the environmental samples (*χ*^2^ = 26.7553, *P* < 0.001). For avian influenza A, chopping board swabs had the highest positive rate at 50.00% (14/28), while all of the crib swabs were found to be negative. Environmental surface swabs also had a relatively higher proportion of avian influenza A at 18%. Subtype H5 was only observed in the poultry and cage swabs (0.22%, 6/2778 and 0.37%, 3/814, respectively). However, most types of samples were positive for H7 or H9, with the exception of the crib swabs. The highest frequency of subtype H7 was found in chopping board swabs at 39.29%. The environmental surface swabs had the highest prevalence of subtype H9. For poultry samples, the prevalence of avian influenza A in anal (14.29%, 2/14), throat (37.5%, 6/16), and feather swabs (42.86%, 6/14) were higher than that in feces (4.02%, 110/2734).

### Risk factors for the prevalence of avian influenza A in backyard poultry

As shown in Table 3, univariate analysis revealed that type of live birds (trend, *χ*^2^ = 6.170, *P* = 0.013) was a risk factor for avian influenza A in backyard poultry, while the sanitary condition of the premises (trend, *χ*^2^ = 7.407, *P* = 0.006) was a protective one. The numbers of live birds reached borderline significance (*P* = 0.07) as a risk factor. In other words, premises with more types of poultry and the worst sanitary conditions had the higher positivity rate for subtype A. As shown in Table 4, the results of multiple logistic regression analysis indicated that the type of live birds (*OR* = 9.381, 95% *CI*: 1.631–53.950) and the sanitary conditions of the premises (*OR* = 0.186, 95% *CI*: 0.047–0.742) were statistically significant. The number of live birds was marginally statistically significant (*OR* = 3.920, 95% *CI*: 1.050–14.631). Only 3 (0.85%) premises were positive for subtype H9, while none were positive for subtype H5 or H7. So, risk factor analysis for subtypes H5, H7, and H9 could not be conducted.

**Table 3:** Univariate analysis of risk factors for avian influenza virus in backyard poultry

Factors	*n*	Influenza A virus		*χ* ^2^	*P*-value
		Negative (%)	Positive (%)		
Polyculture of flocks from various sources					
No	229	221 (96.51)	8 (3.49)	–	0.77^a^
Yes	123	118 (95.93)	5 (4.07)		
Types of live poultry					
1	195	191 (97.95)	4 (2.05)	6.170	0.013^b^
2	135	129 (95.56)	6 (4.44)		
3	22	19 (86.36)	3 (13.64)		
Number of live poultry					
≤ 19	230	225 (97.83)	5 (2.14)	–	0.07^a^
≥ 20	122	114 (93.44)	8 (6.56)		
Type of nesting area					
Enclosed	127	121 (95.28)	6 (4.72)	0.305	0.581
Open	200	193 (96.50)	7 (3.50)		
Nesting area(m^2^)					
≤ 19	161	154 (96.65)	7 (4.35)	0.604	0.437^b^
20–49	105	101 (96.19)	4 (3.81)		
≥ 50	86	84 (97.67)	2 (2.33)		
Type of raising					
Free ranging	200	194 (97.00)	6 (3.00)	1.284	0.257
In captivity	127	120 (94.49)	7 (5.51)		
Clean practice of premises					
No	241	229 (95.02)	12 (4.98)	–	0.197^a^
Yes	82	81 (98.78)	1 (1.22)		
Disinfection practice of premises					
No	291	279 (95.88)	12 (4.12)	–	1.000^a^
Yes	22	32 (9.88)	1 (3.03)		
Sanitary condition of premises					
Poor	131	121 (92.37)	10 (7.63)	7.407	0.006^b^
Average	189	186 (98.41)	3 (1.59)		
Good	8	8 (100)	0 (0.00)		
Flocks vaccination history					
Yes	65	64 (98.46)	1 (1.54)	–	0.477^a^
No	261	249 (95.40)	12 (4.60)		
Human infection(H7N9) identified nearby					
Yes	46	43 (93.48)	3 (6.52)	–	0.405^a^
No	281	271 (96.44)	10 (3.56)		
Sick or dead flock					
Yes	341	329 (96.48)	12 (3.52)	–	0.236^a^
No	7	6 (85.71)	1 (14.29)		
Poultry source					
From market	280	268 (95.71)	12 (4.29)	–	0.481^a^
Not from market	72	71 (98.61)	1 (1.39)		

^a^ Fisher exact test

^b^ Trend chi-squared test

− Not applicable

**Table 4:** Logistic regression analysis of risk factors for avian influenza virus in backyard poultry

Factors	*P-*value	OR	95% CI
Types of live poultry	0.037		
1		1	
2	0.527	1.576	0.385–6.445
3	0.012	9.381	1.631–53.950
Number of live poultry	0.042	3.920	1.050–14.631
Type of raising area	0.847	0.883	0.248–3.138
Type of raising	0.071	3.360	0.901–12.529
Sanitary condition of premises	0.058		
Poor		1	
Average	0.017	0.186	0.047–0.742
Well	0.999	0.000	–
Flocks vaccination history	0.317	2.993	0.350–25.584
Sick or dead flock	0.403	0.321	0.022–4.608
Poultry source	0.671	1.598	0.184–13.879
Constant	0.159	0.004	

## Discussion

It is generally acknowledged that housing live poultry is associated with human AIV infection. LPMs have been highlighted as major sources of AIV transmission from infected birds and contaminated environments to humans. Consequently, risk factors contributing to the spread of AIV have been explored by several groups and many measures have been implemented by governments or LPM administrators to reduce disease outbreaks, such as LPM closure, banning overnight housing of live poultry, rest days, and regular cleaning and disinfection, etc. [25–27]. Compared with LPMs, backyard poultry flocks have relatively limited qualities and types of species, so that AIV contamination was not as serious as that with LPMs [22]. Nevertheless, with the closure of urban LPMs there has been a general uptrend in the proportion of human infections reported in rural areas, where backyard poultry flocks were found to be the main infection source, especially in Zhejiang Province [14–16]. So, monitoring of backyard poultry flocks and the surrounding environments has become a major focus of AIV control and prevention. The results of the present study found a positive rate of avian influenza A at 12.38% at the premises level in Zhejiang Province, which is far lower than that reported in Chile (27%) [28], which could be explained by the larger number of birds sampled in Chile (median number of chicken = 30) than in Zhejiang Province (median number of chicken = 13). Further, backyard poultry in the Chilean study were located in the *El Yali* ecosystem, which is regarded as a high-risk area for AIV. In addition, subtypes H5, H7, and H9 were detected less frequently than in Vietnam, where the prevalence of these subtypes is reportedly 64.29, 0.51, and 6.12%, respectively [29]. A possible reason for this difference might be that all the samples in the Vietnamese study were obtained from birds, while nearly half samples in the present study were environmental.

As expected, the prevalence of AIV in backyard poultry varied across geographical regions of Zhejiang Province. We found there were significant differences in the spatial distribution of positive backyard poultry and LPMs. A previous study reported that AIV-positive LPMs were primarily located in the northern cities, which had large proportions of human infections of H7N9 [19]. However, we were aware that contamination of backyard poultry in eastern and southern cities (Shaoxing, Ningbo and Wenzhou) was more serious than others in this study, which might result from poorer sanitary condition. However, Therefore, to interrupt the transmission of AIV from backyard poultry to humans, preventative measures are needed to focus on the eastern and southern cities. This finding is valuable for policy makers to identify the targeted areas for interventions.

In the present study, a seasonal trend was observed with peaks in January and a relatively high prevalence in August, which was similar to the findings of our previous study of LPMs. However, subtypes H7N9 and H5N1 did not peak in the summer months [2, 30]. Since September 2016, the frequency of H7N9 human infections has suddenly increased throughout China, including Zhejiang Province [31], with most cases occurring in rural areas as a fifth wave [32]. Nevertheless, our study demonstrated that the subtype H7 prevalence in backyard flocks and surrounding environments was significantly lower in January 2017 than in January 2016, suggesting that backyard poultry might not be the main sources for the increase in human infections of H7N9 in the fifth epidemic. According to a recent study, illegal trading of live poultry in rural areas might be partly responsible for this increase [32].

Another important finding of this study was that the prevalence of AIV subtypes was similar in the poultry and environmental samples, with the exception of subtype H7. Hence, our findings imply that AIV infection of poultry could be estimated by examining the environmental circulation of AIV. Samples collected from chopping boards and sewage were largely positive for subtype H7, in accordance with the results of our previous study of LPMs [22]. Although it was reported that biosecurity measures are indispensable tools to mitigate the transmission of diseases in backyard poultry settings, few studies have investigated the characteristics of contaminated environments [33]. Therefore, the data collected in the present study adds evidence to the state of contamination of backyard poultry settings. Moreover, several studies have focused on improving the knowledge, attitudes, and practices of backyard poultry owners to prevent human AI infection. The findings from the present study indicated that the prevalence of AIV subtypes was similar in poultry and in environmental, is recommended to be informed to the trainees so that targeted environment clean and disinfection would be implemented [17, 34, 35].

The results of logistic regression analysis confirmed that the main risk factors for infection of backyard flocks and contamination of environmental settings by avian influenza A were the types of live birds and the sanitary conditions of the premises. Namely, the circulation of AIV could become more severe by a mixture of diverse types of flocks or poor sanitary status of poultry nesting settings. This finding highlights the importance of cleaning and disinfecting of shelters to reduce contamination, which is usually ignored by flock owners. Wang et al. reported that contact of poultry with wild birds, neighboring backyard waterfowl, and purchases from local live bird markets were risk factors for backyard poultry infection in the Poyang lake region of China [36]. Studies conducted in Egypt and the state of Maryland, USA found that housing chickens and waterfowl together was associated with a higher virus prevalence and seroprevalence of subtype H5N1 in backyard poultry [37]. Biswas et al. also supported separating chickens and ducks at night as a protective measure against H7N9 infection of backyard chickens [38]. Therefore, there is a need to foster concern about mixing diverse flocks based on the evidence provided by these studies and our survey. Routine cleaning and disinfection of wet markets are obligatory in some areas, such as Hong Kong and Guangdong Province [25, 39]. However, there are no guidelines or regulations for the cleaning and disinfection of backyard poultry settings, where AIV generally occur as commonly as in LPMs, according to the results of this study. Thus, guidelines for the cleaning and disinfection of backyard settings are urged to improve sanitary conditions. Although the quantity of poultry was also found to be a risk factor for AIV prevalence in backyard flocks, it was marginally statistically significant in this study. This finding is important to construct a model, as the characteristics of the poultry premises (such as sanitary conditions and housing different species together) are more reasonable parameters than the quantity of birds to predict the risk of human infection.

This study has several limitations. First, the field investigation to identify risk factors for AIV infection was conducted in May 2017, outside of the influenza season in Zhejiang Province, which may have resulted in lower AIV positivity rates. Second, this was a cross-sectional survey, so the evidence to identify risk factors was relatively weaker than with an analytic design, such as with a cohort study. Third, the viability of AIV in positive samples was uncertain because the isolates were not cultured for confirmation.

## Conclusions

By consecutive surveillance and field investigations, our study characterized the prevalence of avian influenza A and subtypes in backyard poultry flocks and environments in Zhejiang Province. Our data indicated that eastern and southern cities in Zhejiang Province should be classified as targeted areas for intervention because of their higher prevalence of AIV. Further, environmental contamination was demonstrated to be indicator of poultry infection of avian influenza A. We also found that the type of live birds was a risk factor, while the sanitary condition of the premise was a protective factor against AI contamination. These findings extend the current knowledge of AIV prevalence in backyard poultry in rural areas of Zhejiang Province and offer evidence for interventional policies and strategies to lower human infections in rural areas.

## Additional file

Additional file 1: Multilingual abstracts in the six official working languages of the United Nations. (PDF 637 kb)

Additional file 2: Table S1 Spatial distribution of AIV in different cities. Table S2 Temporal distribution of AIV between May 2016 and May 2017. (DOCX 18 kb)

## Abbreviations

AIV: Avian influenza virus
CDC: Center for Disease Control and Prevention
LPMs: Live poultry markets
