# Supplementary material for: Risk factors for avian influenza virus in backyard poultry flocks and environments in Zhejiang Province, China: a cross-sectional study
Source: Infect Dis Poverty. 2018 Jun 19;7:65. doi: 10.1186/s40249-018-0445-0 (PMC6006748; doi:10.1186/s40249-018-0445-0)
Supplement: Supplementary file 1 — Multilingual abstracts in the six official working languages of the United Nations. (PDF 637 kb) [file 40249_2018_445_MOESM1_ESM.pdf]

Translation of the abstract into the six official working languages of the United Nations

دراسة شاملة لعدة ثقافات حول عوامل خطورة فيروس إنفلونزا الطيور على أسراب الدواجن فى الافنية الخلفية والبيئة في مقاطعة جيجيانغ فى الصين .

جاو جاو وانغ ، وى تشينغ ، جاو يو ، شى لان ليو ، هاى يان ماو ، ان فو تشين

الملخص
مقدمة: لا تزال حالات الإصابات البشرية بفيروس إنفلونزا الطيور AIV مثيرة للقلق للغاية . وعلى الرغم من الإعتقاد بارتباط سوق الدواجن الحية بالعدوى التي تصيب الإنسان، إلا أن هناك بلاغات عن إصابات بإنفلونزا الطيور، خاصة الوباء الخامس والمعروف بـH7N9- أكثر بكثير في المناطق الريفية التي يتم فيها تربية الدواجن. لكن المعلومات المتوفرة بخصوص عدوى الدواجن المتواجدة فى الافنية الخلفية والتلوث في البيئة المحيطة لا زالت محدودة .
أساليب البحث: تم استخدام نظامين من نظم المراقبة ودراسة استقصائية ميدانية بهدف جمع المعلومات والعينات . تم جمع 4538 عينة عن طريق المراقبة و 3171 عينة عن طريق الدراسة الاستقصائية الميدانية فى الفترة ما بين مايو 2015 ومايو 2017 وتمت مقابلة 352 شخص من مالكى الدواجن فى الأفنية الخلفية استبيانيا فى مايو 2017 وذلك لبحث العوامل المؤثرة فى انتشار فيروس A لانفلونزا الطيور (AIV) وفروعه الأخرى . وتم استخدام نظام PCR-RT لفحص الاحماض النووية للفيروسات . كذلك تم استخدام برنامج الكمبيوتر ArcGIS 10.1 لوضع الخرائط . وتم القيام بتحليل التراجع المتعدد واللوجستى لتحديد عوامل خطورة الاصابة بفيروس (AIV)
النتائج : من بين 428 مكان يتواجد فيه الدواجن من التى تمت مراقبتها عن طريق نظام المراقبة تبين أن 53 (12.38%) منها كان يتواجد فيها فيروس الانفلونزا A . تبين أنه 13 عينة (3.39%) من مجموع 352 من العينات التى تم جمعها من أماكن تواجد الدواجن عن طريق الدراسة الاستقصائية الميدانية كانت مصابة بفيروس الانفلونزا A . وكذلك فان انتشار فيروس (AIV) لم يكن موزعا على نحو متساو وفرعه السائد كان مختلفا بين المدن . وتبين انتشار أعلى لفيروس (AIV) فى المدن الشرقية (شوجينغ و نينجبو) والمدن الجنوبية (وينجو) (16.33% , 8.94% و 7.30% عل التوالى) . وكانت أعلى نسبة للاصابة بفروع فيروس AIV خلال شهر يناير وخاصة فى عام 2016 (23.26% , 301/70) ونسبة الاصابة بالفيروس الفرعى H5/H7/H9 كانت 2.53% (4538/115). وكان الفيروس الفرعى H5 هو الأقل انتشارا بينما الفيروسات الفرعية H7 و H9 كانت نسبة الاصابة بهما متشابهة (1.50% و 1.32% , على التوالى) . العينات البيئية وعينات الدواجن احتوت على نسبة متشابهة لانتشار فيروس AIV 4.46% مقابل 5.06% وتعتبر أنواع الطيور الحية من العوامل المسببة للعدوى , والظروف الصحية للمكان من العوامل التى توفر الحماية من الاصابة من فيروس الانفلونزا A
االاستنتاجات : الفيروسات الفرعية لفيروس AIV كانت منتشرة فى قطعان الدواجن فى الأفنية الخلفية والمناطق المحيطة فى مقاطعة جيجانغ . وتعتبر أنواع الطيور الحية من العوامل المسببة للعدوى , والظروف الصحية للمكان من العوامل التى توفر الحماية من الاصابة من فيروس الانفلونزا A . وتلقى هذه النتائج الضوء على خصائص الاصابة بالفيروسات الفرعية لفيروس AIV والتركيز على أهمية الحد من انتشار فيروس AIV فى أماكن تواجد الدواجن فى الأفنية الخلفية .

Translated from English version into Arabic by Msalsa, proofread by Sari_, through

TRANSLATORS
WITHOUT BORDERS

中国浙江省散养禽及其涉禽环境禽流感病毒污染危险因素横断面调查

王笑笑，程伟，余昭，刘社兰，茅海燕，陈恩富

摘要
引言：人感染禽流感受到广泛关注。尽管活禽市场被认为与病例相关，但是越来越多的病例报告显示与散养禽有关，尤其是第五个流行季。但是目前关于散养禽的禽流感病毒感染情况，以及其涉禽外环境的污染状况所知甚少。
方法：通过浙江省禽流感监测系统和专题现场调查收集数据和标本。2015 年 5 月-2017 年 5 月，通过监测系统共收集 4538 份标本；通过专题现场调查，对 352 户散养禽户开展了问卷调查，并收集了 3171 份标本，以了解影响禽流感病毒污染的因素。用 RT-PCR 对标本进行禽流感病毒核酸检测。用 ArcGIS 10.1 软件绘制地图。用单因素和多因素回归模型分析禽流感病毒污染的危险因素。
结果：监测系统所监测的 428 户散养禽户中，有 53 户（12.38%）为甲型阳性。专题现场调查的 352 户中，有 13 户（3.39%）为甲型阳性。禽流感病毒污染阳性率和亚型的阳性率地区分布不均衡。东部（绍兴和宁波）和南部（温州）的甲型阳性率较高，分别为 16.33%、8.94%和 7.30%。1 月份的污染最严重，尤其是 2016 年 1 月（23.26%, 70/301）。H5/H7/H9 的阳性率为 2.53% (115/4538)。H5 亚型的阳性率最低，H5 和 H9 的阳性率相近（分别为 1.50%和 1.32%）。散养禽的禽流感病毒感染率和外环境的病毒污染率相近（分别为 4.46%和 5.06%）。活禽种类数是禽流感病毒污染的危险因素，外环境卫生状况则是保护因素。
结论：浙江省的散养禽及其涉禽外环境禽流感病毒污染普遍。活禽种类数和外环境卫生状况是禽流感病毒污染的影响因素。上述发现揭示了浙江省散养禽及其外环境不同亚型禽流感病毒的污染特征，提示应减少散养禽及其外环境的病毒污染。

Translated from English version into Chinese by Xiao-Xiao Wang

Facteurs de risque du virus de la grippe aviaire dans les élevages de volailles de basse-cour et l' environnement de la province de Zhejiang, Chine : étude transversale.

Xiao-Xiao Wang, Wei Cheng, Zhao Yu, She-Lan Liu, Hai-Yan Mao, En-Fu Chen

Résumé
Contexte: La transmission humaine du virus de la grippe aviaire reste une préoccupation grave. On associe les marchés aux volailles vivantes à la transmission du virus mais des cas encore plus nombreux d’infection humaine ont été rapportés dans les zones rurales où se pratique l’élevage en basse-cour, notamment lors de la cinquième épidémie de H7N9. Toutefois, on dispose de peu d'informations sur l'infection des volailles de basse-cour et la contamination de l'environnement aux alentours.
Méthodes: Deux systèmes de surveillance et un relevé de terrain ont été utilisés pour recueillir des données et des échantillons. Au total, 4 538 échantillons ont été prélevés par la surveillance et 3 171 ont été relevés sur le terrain entre mai 2015 et mai 2017; 352 propriétaires de basses-cours ont été interrogés en mai 2017 au moyen d’un questionnaire, afin d’étudier les facteurs influant sur la prévalence du virus de la grippe aviaire de type A et des autres sous-types. Les acides nucléiques des virus ont été testés par TI-RCP. Nous avons utilisé le logiciel ArcGIS 10.1 pour générer des cartes. Des analyses de régression logistique et univariées ont été réalisées afin d’identifier les facteurs de risque d’infection par le virus de la grippe aviaire.

Résultats: Sur les 428 basses-cours surveillées, 53 (12,38 %) étaient positives pour le virus de la grippe aviaire de type A. Sur les 352 échantillons du relevé de terrain, 13 (3,39 %) étaient positifs. La prévalence du virus de la grippe aviaire était répartie de manière inégale et le sous-type dominant variait d’ une ville à l’ autre. Les villes de l’ est (Shaoxing et Ningbo) et du sud (Wenzhou) de la province présentaient une plus forte prévalence du virus (16,33 %, 8,94 % et 7,30 % respectivement). La contamination par les sous-types du virus a été particulièrement grave en janvier, notamment en 2016 (23,26 %, 70 sur 301). Le taux de positivité aux sous-types H5, H7 et H9 était de 2,53 % (115 sur 4 538). Le sous-type H5 était le moins répandu, tandis que H7 et H9 donnaient des taux de positivité similaires (1,50 % et 1,32 %, respectivement). Les échantillons des basses-cours et environnementaux présentaient une prévalence similaire du virus (4,46 % contre 5,06 %). Le type d’ oiseaux vivants était un facteur de risque et l’ état sanitaire de l'endroit était un facteur de protection contre la contamination par le virus de la grippe aviaire de type A.

Conclusions: Des sous-types du virus de la grippe aviaire étaient très répandus dans les basses-cours et les alentours de la province du Zhejiang. Les types d’oiseaux vivants et les conditions sanitaires locales étaient associés à la contamination par ce virus. Ces résultats mettent en lumière les caractéristiques de la contamination par les sous-types du virus de la grippe aviaire et l’importance de réduire la circulation du virus dans les basses-cours.

Translated from English version into French by Suzanne Assenat, proofread by Karine H, through

TRANSLATORS
WITHOUT BORDERS

Факторы риска вируса птичьего гриппа домашних птиц на частных подворьях и в природе в провинции Чжэцзян в Китае: перекрёстное исследование

Сяо-Сяо Ван, Вэй Чэн, Чжао Юй, Шэ-Лань Лю, Хай-Янь Мао, Энь-Фу Чэнь

Аннотация

Справочная информация: Заражение человека вирусом птичьего гриппа (AIV) по-прежнему вызывает беспокойство. Несмотря на то, что деятельность рынков по реализации живой птицы, считается связанной с человеческими инфекциями, всё больше случаев инфицирования регистрируется в районах содержания домашних птиц на частных подворьях, особенно в случае пятой эпидемии гриппа A (H7N9). Вместе с тем, о заражении домашних птиц на частных подворьях и заражении прилегающей окружающей среды имеются лишь ограниченные сведения.

Методы: Сбор данных и образцов осуществлялся с помощью двух систем наблюдения и методом полевого исследования. В целом, с мая 2015 года по май 2017 года было получено 4538 образцов путём наблюдения и 3171 образец методом полевого исследования, вместе с тем, в мае 2017 года с целью исследования факторов, влияющих на инфицирование вирусом птичьего гриппа A (AIV) и другими подтипами этого вируса гриппа методом анкетирования было

опрошено 352 владельца частных подворьев. Для анализа нуклеиновых кислот вирусов использовалась ПЦР с обратной транскрипцией (ОТ-ПЦР). Для построения карт было задействовано геоинформационное программное обеспечение ArcGIS 10.1. С целью выявления факторов риска инфицирования вирусом птичьего гриппа был проведён одномерный и логистический регрессионный анализ.

Результаты: Из 428 мест содержания домашних птиц, по которым проводился обзор при помощи системы наблюдения, в 53 (12,38%) случаях был обнаружен вирус гриппа А. Из 352 образцов, взятых на местах содержания домашних птиц в результате проведённого полевого исследования, в 13 (3,39%) случаях был выявлен вирус гриппа А. Наблюдалось неравномерное распространение вируса птичьего гриппа и доминантного подтипа вируса по городам. В восточных (Шаосин и Нинбо) и в южных городах (Вэньчжоу) наблюдался высокий уровень инфицирования вирусом птичьего гриппа (16,33%; 8,94%, и 7,30% соответственно). Самый серьёзный уровень инфицирования подтипами вируса птичьего гриппа приходился на январь, особенно в 2016 году (23,26%; 70/301). Уровень положительных проб на подтипы вируса гриппа H5/H7/H9 составил 2,53% (115/4538). Наименее распространённым оказался подтип вируса гриппа H5, тогда как подтипы H7 и H9 обладали сопоставимыми уровнями положительных случаев (1,50% и 1,32%, соответственно). Образцы, полученные по поголовью домашних птиц и из окружающей среды, содержали аналогичный уровень инфицирования вирусом птичьего гриппа (4,46% против 5,06%). Вид живых птиц являлся фактором риска, а санитарные условия содержания являлись защитным фактором против заражения птичьим гриппом А.

Выводы: Подтипы вируса птичьего гриппа преобладали как в поголовье домашних птиц на частных подворьях, так и в окружающей среде в провинции Чжэцзян. Риск заражения вирусом гриппа А был связан с видом живой птицы, а также с санитарными условиями окружающей среды. Данные выводы проливают свет на характеристики заражения подтипами вируса птичьего гриппа и подчёркивают важность снижения циркуляции вируса птичьего гриппа среди домашних птиц на частных подворьях.

Translated from English version into Russian by Liudmila Tomanek, proofread by Tatiana Kary, through

Factores de riesgo por el virus de la gripe aviar en aves de corral y entornos de desarrollo de la provincia de Zhejiang, China: un estudio transversal

Xiao-Xiao Wang, Wei Cheng, Zhao Yu, She-Lan Liu, Hai-Yan Mao, En-Fu Chen

Resumen

Antecedentes: La infección humana por el virus de la gripe aviar (AIV, por sus siglas en inglés) sigue causando gran preocupación. Aunque se cree que los mercados de aves de corral vivas están asociados

con infecciones humanas, cada vez se han informado más infecciones en áreas rurales con aves de corral, especialmente en la quinta epidemia de H7N9. Sin embargo, se dispone de escasa información sobre la infección de aves de corral y la contaminación del medio ambiente circundante.

Métodos: Se utilizaron dos sistemas de vigilancia y un estudio sobre el terreno para recabar datos y tomar muestras. En total, se tomaron 4538 muestras a través del sistema de vigilancia, y 3171 a través del estudio sobre el terreno entre mayo de 2015 y mayo de 2017, mientras que, en mayo de 2017, se entrevistó a 352 dueños de aves de corral por medio de un cuestionario para investigar los factores que influyen en la prevalencia del virus de la gripe aviar A y de otros subtipos de AIV. Se empleó la reacción en cadena de la polimerasa con transcriptasa inversa (RT-PCR, por sus siglas en inglés) para analizar los ácidos nucleicos de los virus. Se utilizó el programa ArcGIS 10.1 para generar mapas. Se realizaron análisis univariantes y de regresión logística para identificar los factores de riesgo por la infección por AIV.

Resultados: De los 428 terrenos con aves de corral que se observaron a través del sistema de vigilancia, 53 (12,38 %) arrojaron resultados positivos para el virus de la gripe A. De las 352 muestras obtenidas en los terrenos con aves de corral que se observaron a través del estudio sobre el terreno, 13 (3,39 %) arrojaron resultados positivos para el virus de gripe A. La prevalencia de AIV se distribuyó de manera desigual, y el subtipo predominante difirió de ciudad en ciudad. Las ciudades orientales (Shaoxing y Ningbo) y las meridionales (Wenzhou) presentaron una prevalencia superior de AIV (16,33 %, 8,94 % y 7,30 %, respectivamente). La contaminación de los subtipos de AIV resultó más grave en enero, en especial en 2016 (23,26 %, 70/301). La tasa positiva del subtipo H5/H7/H9 fue de 2,53 % (115/4538). El subtipo H5 fue el menos frecuente, mientras que los subtipos H7 y H9 arrojaron tasas positivas similares (1,50 % y 1,32 %, respectivamente). Las muestras de aves de corral y de los entornos de desarrollo mostraron una prevalencia similar de AIV (4,46 % versus 5,06 %). El tipo de aves vivas fue un factor de riesgo, y el estado sanitario del entorno fue un factor de protección contra la contaminación de la gripe A.

Conclusiones: Los subtipos de AIV fueron predominantes en las aves de corral y los entornos de desarrollo circundantes de la provincia de Zhejiang. Los tipos de aves vivas y el estado sanitario del ambiente de desarrollo fueron asociados con la contaminación de la gripe A. Estos resultados arrojan luz sobre las características de la contaminación de los subtipos de AIV y hacen hincapié en la importancia de reducir la circulación de AIV en los entornos de aves de corral.

Translated from English version into Spanish by María Florencia Blanco Cabrera, proofread by Marilyn Santiago, through

TRANSLATORS
WITHOUT BORDERS
