# Supplementary material for: Risk factors for avian influenza virus in backyard poultry flocks and environments in Zhejiang Province, China: a cross-sectional study
Source: Infect Dis Poverty. 2018 Jun 19;7:65. doi: 10.1186/s40249-018-0445-0 (PMC6006748; doi:10.1186/s40249-018-0445-0)
Supplement: Supplementary file 2 — Table S1 Spatial distribution of AIV in different cities. Table S2 Temporal distribution of AIV between May 2016 and May 2017. (DOCX 18 kb) [file 40249_2018_445_MOESM2_ESM.docx]

Supplements

Table 1 Spatial distribution of AIV in different cities.

| City | Sum | Positive (%) | | | | |
| --- | --- | --- | --- | --- | --- | --- |
|  |  | A | H5 | H7 | H9 | H5/H7/H9 |
| Hangzhou | 578 | 14(2.42) | 0(0) | 0(0) | 0(0) | 0(0) |
| Huzhou | 312 | 3(0.96) | 0(0) | 1(0.32) | 2(0.64) | 2(0.64) |
| Jiaxing | 323 | 1(0.31) | 0(0) | 1(0.31) | 0(0) | 1(0.31) |
| Jinhua | 847 | 39(4.60) | 4(0.47) | 6(0.71) | 17(2.01) | 24(2.83) |
| Lishui | 482 | 26(5.39) | 2(0.41) | 5(1.04) | 0(0) | 7(1.45) |
| Ningbo | 179 | 16(8.94) | 1(0.56) | 4(2.23) | 0(0) | 5(2.79) |
| Quzhou | 348 | 10(2.87) | 0(0) | 0(0) | 6(1.72) | 6(1.72) |
| Shaoxing | 398 | 65(16.33) | 2(0.50) | 32(8.04) | 34(8.54) | 50(12.56) |
| Taizhou | 261 | 12(4.60) | 0(0) | 2(0.77) | 0(0) | 2(0.77) |
| Wenzhou | 370 | 27(7.30) | 0(0) | 17(4.59) | 1(0.27) | 18(4.86) |
| Zhoushan | 440 | 0(0) | 0(0) | 0(0) | 0(0) | 0(0) |
| Total | 4538 | 213(4.69) | 9(0.20) | 68(1.50) | 60(1.32) | 115(2.53) |

Table 2 Temporal distribution of AIV between May 2016 and May 2017

| Year | Month | Sum | Positive (%) | | | | |
| --- | --- | --- | --- | --- | --- | --- | --- |
|  |  |  | A | H5 | H7 | H9 | H5/H7/H9 |
| 2015 | 5 | 87 | 8(9.20) | 0(0) | 1(1.15) | 0(0) | 1(1.15) |
|  | 6 | 134 | 1(0.75) | 0(0) | 1(0.75) | 1(0.75) | 1(0.75) |
|  | 7 | 84 | 3(3.57) | 0(0) | 0(0) | 2(2.38) | 2(2.38) |
|  | 8 | 62 | 0(0) | 0(0) | 0(0) | 0(0) | 0(0) |
|  | 9 | 148 | 2(1.35) | 0(0) | 0(0) | 0(0) | 0(0) |
|  | 10 | 183 | 0(0) | 0(0) | 0(0) | 0(0) | 0(0) |
|  | 11 | 87 | 2(2.30) | 0(0) | 0(0) | 1(1.15) | 1(1.15) |
|  | 12 | 198 | 0(0) | 0(0) | 0(0) | 0(0) | 0(0) |
| 2016 | 1 | 301 | 70(23.26) | 2(0.66) | 42(13.95) | 21(6.98) | 54(17.94) |
|  | 2 | 260 | 2(0.77) | 0(0) | 0(0) | 0(0) | 0(0) |
|  | 3 | 259 | 8(3.09) | 0(0) | 0(0) | 6(2.32) | 6(2.32) |
|  | 4 | 63 | 0(0) | 0(0) | 0(0) | 0(0) | 0(0) |
|  | 5 | 29 | 0(0) | 0(0) | 0(0) | 0(0) | 0(0) |
|  | 6 | 146 | 1(0.68) | 0(0) | 0(0) | 0(0) | 0(0) |
|  | 7 | 82 | 0(0) | 0(0) | 0(0) | 0(0) | 0(0) |
|  | 8 | 94 | 10(10.64) | 4(4.26) | 0(0) | 1(1.06) | 4(4.26) |
|  | 9 | 131 | 7(5.34) | 0(0) | 0(0) | 4(3.05) | 4(3.05) |
|  | 10 | 180 | 0(0) | 0(0) | 0(0) | 0(0) | 0(0) |
|  | 11 | 265 | 7(2.64) | 0(0) | 1(0.38) | 2(0.75) | 3(1.13) |
|  | 12 | 328 | 23(7.01) | 0(0) | 5(1.52) | 14(4.27) | 15(4.57) |
| 2017 | 1 | 261 | 30(11.49) | 2(0.77) | 15(5.75) | 4(1.53) | 19(7.28) |
|  | 2 | 421 | 15(3.56) | 0(0) | 0(0) | 0(0) | 0(0) |
|  | 3 | 402 | 8(1.99) | 0(0) | 0(0) | 0(0) | 0(0) |
|  | 4 | 181 | 8(4.42) | 1(0.55) | 3(1.66) | 3(1.66) | 4(2.21) |
|  | 5 | 152 | 8(5.26) | 0(0) | 0(0) | 1(0.66) | 1(0.66) |
| Total | | 4538 | 213(4.69) | 9(0.20) | 68(1.50) | 60(1.32) | 115(2.53) |
